# Complex Movement Disorders in Ataxia with Oculomotor Apraxia Type 1: Beyond the Cerebellar Syndrome

**DOI:** 10.5334/tohm.557

**Published:** 2020-10-07

**Authors:** José Luiz Pedroso, Thiago Cardoso Vale, Sophia Caldas Gonzaga da Costa, Mariana Santos, Isabel Alonso, Orlando Graziani Povoas Barsottini

**Affiliations:** 1 Ataxia Unit, Department of Neurology, Universidade Federal de São Paulo, Sao Paulo, BR; 2 Movement Disorders Unit, Departament of Internal Medicine, Universidade Federal de Juiz de Fora, Juiz de Fora, BR; 3 Instituto de Investigação e Inovação em Saúde, Universidade do Porto, Porto, PT

**Keywords:** ataxia, ataxia with oculomotor apraxia, ataxia with oculomotor apraxia type 1, movement disorders, cerebellum

## Abstract

**Background:**

Ataxia with oculomotor apraxia (AOA1) is characterized by early-onset progressive cerebellar ataxia with peripheral neuropathy, oculomotor apraxia and hypoalbuminemia and hypercholesterolemia.

**Case Report:**

A 23-year-old previously healthy woman presented with slowly-progressive gait impairment since the age of six years. Neurological examination revealed profound areflexia, chorea, generalized dystonia and oculomotor apraxia. Brain MRI revealed mild cerebellar atrophy and needle EMG showed axonal sensorimotor neuropathy. Whole exome sequencing revealed a mutation in the aprataxin gene.

**Discussion:**

AOA1 can present with choreoathetosis mixed with dystonic features, resembling ataxia-telangiectasia. This case is instructive since mixed and complex movement disorders is not very common in AOA1.

**Highlights:**

- Ataxia with oculomotor apraxia type 1 (AOA1) is characterized by early-onset ataxia and oculomotor apraxia caused by variants in the APTX gene.
- Ataxia is usually not the sole movement abnormality in AOA1.
- Hyperkinetic movement disorders, especially chorea and dystonia, may occur.
- Mixed and complex movement disorders is not very common in AOA1.
- Patients with early-onset ataxia associated with mixed movement disorders should also be investigated for AOA1.

## Introduction

Autosomal recessive cerebellar ataxias (ARCA) are a heterogeneous group of diseases. Ataxia with oculomotor apraxia (AOA) is a genetic condition characterized by progressive cerebellar ataxia and oculomotor apraxia, and the most common AOA are subtypes 1, 2 and 4 [1]. AOA1 (MIM208920) usually presents with early onset, progressive ataxia, peripheral neuropathy and oculomotor apraxia, accompanied by hypoalbuminemia and hypercholesterolemia [1]. The disease is caused by variants in the *APTX* gene, and the locus is on chromosome 9p13 [2]. The *APTX* gene encodes aprataxin, a protein that has a suggested role in DNA break repair, largely expressed in the cerebellum, basal ganglia, cerebral cortex and spinal cord. To date, several missense, nonsense and frameshift mutations have been identified, mostly in Europe and Japan.

Ataxia is usually not the sole movement abnormality in AOA1. Hyperkinetic movement disorders, particularly chorea and dystonia, may also occur [3]. We report a case of a complex and mixed movement disorders in a patient with AOA1, expanding the phenotype beyond the cerebellar syndrome.

**Figure 1:**
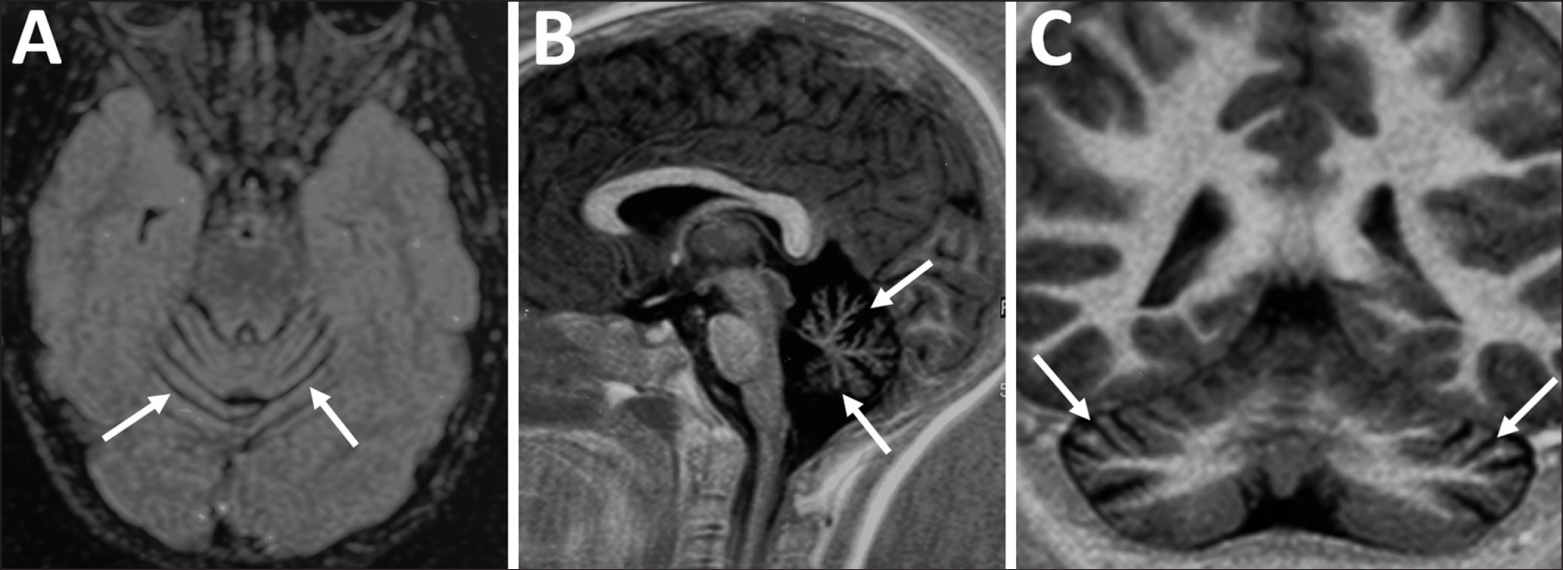
Axial Flair (panel A), sagittal T1-weighted (panel B) and coronal T1-weighted (panel C) brain magnetic resonance imaging showing cerebellar atrophy (white arrows).

A 23-year-old woman, born from consanguineous parents, presented with slowly progressive gait impairment. She normally achieved the developmental milestones until the beginning of the motor symptoms at the age of six years-old. A younger sister was not affected and family history was unremarkable. From six to 15 years-old, the patient complained mostly of loss of balance. For the last eight years, parents observed progressively worsened motor restlessness and abnormal posture of the limbs. During the last two years, there were prominent worsening of gait and abnormal movements. Neurological examination was characterized by ataxia, chorea, dystonia, myoclonic jerks and oculomotor apraxia (Video 1). There was decreased deep tendon reflexes and neuropsychological tests were normal. Serum alpha-fetoprotein (AFP) and albumin levels were normal (albumin serum level was 4.3 g/dl; normal albumin range: 3.4 to 5.4 g/dl]), but there was a mild increase in cholesterol levels (203 mg/dl; normal levels: below 200 mg/dl). Brain magnetic resonance imaging performed two years after the beginning of symptoms showed cerebellar atrophy (Figure 1), and needle electromyographic studies showed lower-limb chronic axonal sensorimotor neuropathy. A genetic panel sequencing for AOA including *APTX, SETX* (senataxin), *PIK3R5, PNPK* genes was performed, and disclosed a pathogenic homozygous variant in the *APTX* (c.[837G>A];[837G>A]) gene, confirming the diagnosis of AOA1. The patient was treated with trihexyphenidyl 8 mg daily and clonazepam 2 mg daily with mild improvement. There was no improvement with levodopa and botulinum toxin injections.

**Video 1:** **Complex movement disorders in ataxia with oculomotor apraxia type 1.** Segment one shows an ataxic gait pattern with generalized dystonia (affecting predominantly the trunk and cervical region), myoclonic jerks and choreoathetotic movements in her hands. Segment two shows oculomotor apraxia with hypometric saccades and cervical dystonia with choreoathetotic movements in her hands. Segment three shows cervical, upper and lower limb dystonic postures with choreoathetotic movements in her upper-limbs and dysmetria in the finger-to-nose maneuver.

Hyperkinetic movement disorders occur in about one-third of patients with ARCA [3]. Involuntary movements occur in the majority of patients with ataxia-telangiectasia and AOA1 [4], ranging from mild to severe abnormal movements. When initially present, it poses significant challenges to the accurate diagnosis. Chorea is reported to be present in between 40 to 80% of patients [5] at the beginning of the illness and is usually most severe in patients with early onset [6]. It can affect the face, laryngo-pharynx and limbs. Dystonia is present between 25 to 50% of patients after the disease onset or several years later [7]. In our patient, it was very difficult to differentiate between myoclonus or dystonic jerks.

AOA1 typically manifests with gait ataxia in the first decade of life, followed by dysarthria and upper limb dysmetria. Axonal sensorimotor neuropathy ultimately leading to distal amyotrophy occurs in the majority of patients [8]. Oculomotor apraxia is also very frequent, being present in 86% of patients [5]. The main differential diagnosis for patients who present with early onset ataxia and chorea include ataxia telangiectasia (AT), *MRE11A* gene mutations (AT-like) and AOA1. Low serum concentrations of albumin and raised serum concentration of total cholesterol are found in 83% and 75% of individuals with disease duration of more than ten to 15 years. Normal levels of albumin, particularly at the beginning of the disease, can occur [9].

In the largest international cohort published to date, involving 80 patients with AOA1, proportions of chorea, dystonia and myoclonus were of 40%, 25% and 8%, respectively [9]. It was similar to the proportions seen in the study by Yokoseki et al. [10] in a Japanese population. Parkinsonism was seen in only 3% of the population and intellectual disability, predominantly seen in Japanese patients, was detected in 53% of the population of this international cohort.

The main findings on brain pathology of ARCA patients, including those with AOA1, are marked loss of cerebellar Purkinje cells [4]. The pathophysiological basis of the involuntary movements remains controversial, but is probably linked to basal ganglia damage, though some evidence points out a cerebellar origin for chorea and dystonia. Disynaptic pathways connecting the dentate nucleus and striatum as well as the subthalamic nucleus and cerebellar cortex may be damaged [4].

This case is very instructive since mixed and complex movement disorders is not very common in AOA1. Early recognition of hyperkinetic movement disorders in AOA1 is relevant for an appropriate symptomatic treatment. And finally, patients with early onset ataxia associated with mixed movement disorders should be genetically investigated for several disease, including AOA1.

## Author Contribuction

1. Case report project: A. Conception, B. Organization, C. Execution
2. Data collection: A. Case Report
3. Manuscript: A. Writing of the first draft, B. Review and Critique.
  Pedroso JL: 1A, 1B, 1C, 3A, 3B.
  Vale TC: 1A, 1B, 1C, 3A, 3B.
  Costa SCG: 1A, 1B, 1C.
  Santos M: 2A, 3B.
  Alonso I: 2A, 3A, 3B.
  Barsottini OGP: 1A, 1B, 1C, 3A, 3B.
